# Preterm Birth Therapies to Target Inflammation

**DOI:** 10.1002/jcph.2107

**Published:** 2022-09-15

**Authors:** Ioannis Pavlidis, Sarah J. Stock

**Affiliations:** ^1^ University of Warwick Biomedical Research Unit in Reproductive Health Coventry UK; ^2^ University of Edinburgh Usher Institute Edinburgh UK

**Keywords:** inflammation, obstetrics, preterm birth, therapeutics, women's health

## Abstract

Preterm birth (PTB; defined as delivery before 37 weeks of pregnancy) is the leading cause of morbidity and mortality in infants and children aged <5 years, conferring potentially devastating short‐ and long‐term complications. Despite extensive research in the field, there is currently a paucity of medications available for PTB prevention and treatment. Over the past few decades, inflammation in gestational tissues has emerged at the forefront of PTB pathophysiology. Even in the absence of infection, inflammation alone can prematurely activate the main components of parturition resulting in uterine contractions, cervical ripening and dilatation, membrane rupture, and subsequent PTB. Mechanistic studies have identified critical elements of the complex inflammatory molecular pathways involved in PTB. Here, we discuss therapeutic options that target such key mediators with an aim to prevent, postpone, or treat PTB. We provide an overview of more traditional therapies that are currently used or being tested in humans, and we highlight recent advances in preclinical studies introducing novel approaches with therapeutic potential. We conclude that urgent collaborative action is required to address the unmet need of developing effective strategies to tackle the challenge of PTB and its complications.

According to the long‐standing definition by the World Health Organization, preterm birth (PTB) is delivery before 37 completed weeks of gestation. It represents 5% to 18% of deliveries worldwide, with the impact being higher in low‐ and middle‐income countries.^1^ This amounts to a total of around 15 million preterm births each year.^1^ Prematurity‐related complications can confer significant neonatal morbidity with potential long‐term implications. These involve adverse neurological outcomes including cerebral palsy, neurodevelopmental delay, vision, and hearing impairment; adverse respiratory outcomes including bronchopulmonary dysplasia; and a series of adverse outcomes that can affect different systems resulting in lifelong disabilities.^2^ Every year there are about 1 million neonatal deaths attributed directly to these complications, with an estimated cost of more than $26 billion to the US economy,^3^ more than £3 billion to the UK economy,^4^ and more than AU$2 million to the Australian economy^5^ per annum. The above collectively constitute PTB as the leading cause of neonatal morbidity and mortality.^2^ This further extends to children aged <5 years. In a recent report, complications of PTB were the leading cause of mortality in this age group, accounting for virtually 20% of all deaths.^6^ In 2015, the United Nations produced a series of Sustainable Development Goals as a framework for world development and set out specific targets to address these goals. To achieve goal 3, which is to “ensure healthy lives and promote well‐being for all at all ages,” one of the targets is to reduce mortality of children aged <5 years to 25 per 1000 live births from the current rate of 38 per 1000. With PTB being the most common reason for this mortality rate, it is now recognized that to achieve this goal, it is crucial to improve our ability to predict, prevent, and treat PTB.

The substantial health and economic burden of PTB makes the development of effective prevention and treatment strategies imperative. However, there has been limited success in the field despite ongoing efforts for more than 60 years. The majority of medications used in PTB therapeutics are not pregnancy‐specific and have been repurposed from their use in different conditions. In addition, most phase II/III clinical trials to date have reported negative results on the efficacy of these drugs to prevent/prolong PTB. This is often attributed to the fact that PTB is considered as a multifactorial condition making it difficult for single preventative/treatment options to be effective across all cases. Yet, it is also reflective of our current incomplete understanding of the underlying pathophysiology of PTB. Compelling evidence accumulated over the past 3 decades has placed intrauterine inflammation in the epicenter of the complex mechanistic pathways involved in spontaneous PTB as well as in normal term labor.^7^

Parturition is a highly coordinated process characterized by fundally dominant synchronized uterine contractions, ripening, and dilatation of the cervix and remodeling and rupture of the fetal membranes. Features of inflammation have been described as playing an important role in these events.^8^ Specifically, during term labor, immune cells including neutrophils and macrophages infiltrate the uterus, the cervix, and the fetal membranes.^9^ The initial driver of this inflammatory response is thought to be signals of adequate maturation of the fetus, including the increased production of surfactant protein A and phospholipids by the fetal lung.^10^ This is accompanied by an increase in the expression of proinflammatory cytokines and chemokines including tumor necrosis factor‐α (TNFα),^11^ interleukin (IL)‐1β,^12^ IL‐6,^13^ and IL‐8^14^ in gestational tissues including the placenta. These mediators can both stimulate and amplify the expression of prostaglandins and matrix metalloproteinases. The former are known potent stimulators of myometrial contractions, while both can trigger degradation of the extracellular matrix, resulting in fetal membrane rupture and cervical ripening.^15,16^ This pathway can be untimely activated following infection; rather than fetal maturation, activation can be triggered either by intrauterine infection or systemic infection.^17^ In fact, infection is currently considered the most common cause of PTB, especially before 28 weeks, accounting for an estimated 40% of cases.^18^ Although several anatomic and immunologic barriers aim at protecting in utero bacterial colonization, the amniotic compartment is not intrinsically sterile and bacteria can invade the cavity, most commonly via an ascending route from the vagina.^19^ In the event of a microbial invasion of the amniotic cavity, specialized innate pattern recognition receptors named toll‐like receptors (TLRs) that are expressed in gestational tissues recognize specific microbial parts triggering a cascade of molecular events.^20^ These culminate in the increased expression of the above‐mentioned cytokines and chemokines through the regulatory function of the transcription factors nuclear factor kappa B (NFkB),^21^ activator protein‐1^22^ and p38 mitogen‐activated protein kinase (MAPK).^23^ Despite similarities in the type of immune cells and cytokines/chemokines involved, the subsequent inflammatory response following infection‐mediated preterm labor is considered to be more robust compared to normal term labor without an infectious trigger.^24^

Given the pivotal role of the inflammatory response in PTB pathophysiology, targeting inflammation is an important part in the design of effective therapeutic approaches. These can take different forms depending on the objectives of each strategy: Prevention strategies typically aim at reducing the incidence of PTB in high‐risk women; fetal optimization strategies aim at briefly delaying progression of PTB once preterm labor has already started to allow for medication aiming at fetal lung maturation and neuroprotection to be administered; finally, treatment strategies aim at regulating the timing of delivery by prolonging gestation when this is thought to be beneficial for the fetus. This review summarizes a number of options that are readily available or being studied at the moment in PTB therapeutics that at least partly target inflammation. We discuss both more established treatments that are currently administered in humans and more experimental concepts that are being evaluated in preclinical studies. These therapies along with their proposed mechanism of targeting inflammation are summarized in Table 1.

## Progestogens

Progestogens have a long‐standing history in PTB therapeutics. Progesterone (P4) was one of the first drugs to be tested for PTB prevention 60 years ago, and synthetic progesterone analogs are still used to this day as part of the most well‐studied PTB prevention strategies.^25^ During pregnancy, progesterone exerts multiple actions in gestational tissues with a cumulative effect of maintaining the ongoing pregnancy. In the myometrium, it promotes quiescence by suppressing the expression of procontractile genes, most notably Connexin‐43.^26^ In addition, P4 has an anti‐inflammatory effect in myometrial cells, where it reduces the expression of cytokines such as IL‐1β and chemokines such as IL‐8).^27^ Importantly, P4 acts to modulate prostaglandin levels by inhibiting cyclooxygenase (COX)‐2 expression via the progesterone receptor'santagonizing action against NFkB.^27^ This effect has also been shown to extend in the fetal membranes, likely preventing their inflammation‐mediated premature remodeling and subsequent rupture.^28^ A similar prostaglandin‐regulating anti‐inflammatory mechanism has also been proposed to regulate cervical remodeling, expression of adhesion molecules, and subsequent ripening of the cervix.^29,30^

Due to these effects of progestogens on all different components of parturition, their use as prophylactic agents in PTB prevention has been extensively studied in the past 20 years. The rationale of use includes identifying women at high risk of PTB and administering progesterone preparations throughout pregnancy in an attempt to reduce the incidence of PTB and mitigate related complications. Oral, intramuscular, and vaginal administration of progestogens have been tested. Encouragingly, the most recent systematic review and meta‐analysis of randomized trials has reported that vaginal P4 and intramuscular 17‐hydroxyprogesterone caproate (17‐OHPC), but not oral P4, appear to be reducing the incidence of PTB before 34 weeks in high‐risk singleton pregnancies, as identified by history of previous PTB or a sonographically short cervix.^31^ Notably, 17‐OHPC has been the only medication approved by the US Food and Drug Administration (FDA) in 2011 for use in PTB prevention since it was first shown to reduce the incidence of preterm delivery in high‐risk women.^32^ However, there is still no consensus on the use of progestogens in PTB prevention as several trials have announced mixed findings. Indeed, the largest randomized controlled trials (RCTs) published to date have shown no difference in PTB rates with the use of either vaginal P4 (OPPTIMUM trial)^33^ or intramuscular 17‐OHPC (PROLONG trial).^34^ This latter study was pivotal in the FDA's decision to withdraw the approval of 17‐OHPC in 2020.^35^ Still, the most recent systematic review and meta‐analysis (EPPPIC study) concluded that both P4 and 17‐OHPC reduced the incidence of PTB before 34 weeks in high‐risk pregnancies.^31^ While this did not impact the FDA's decision, both the American College of Obstetricians and Gynecologists^36^ and the Society for Maternal‐Fetal Medicine^37^ currently recommend offering progestogens to this high‐risk population.

When it comes to the route of administration, no difference in the incidence of PTB has been reported between vaginal and intramuscular administration. However, the intramuscular route has been associated with higher occurrence of adverse reactions.^38^ These can include injection site reaction and localized pruritus due to the injection itself, along with nausea, vomiting, headaches, and hot flushes secondary to the systemic administration.^38^ Nevertheless, these are still considered as minor side effects. This is reflective of a major advantage of progestogens as a treatment in pregnancy, which is their favorable safety profile. Critically, this also seems to be the case for the offspring of the mothers that have been exposed to progestogens while in utero.^39,40^ Most of these studies have followed up the offspring until the age of 2 to 6 years.^41^ While the debate on the efficacy of progestogens for PTB prevention is ongoing, the absence of significant maternal of neonatal side effects is a major factor contributing to their widespread use between clinicians and acceptance by the patients. Still, a recent systematic review has suggested a potential association between the use of 17‐OHPC, but not vaginal P4, with increased risk of gestational diabetes mellitus.^42^ In addition, there is a paucity of data on the impact of progestogens during later development. Thus, it is important for future studies to evaluate for potential effects beyond this age and into adolescence and adulthood.

Overall, and despite some conflicting evidence, the use of progestogens in PTB preventive strategies is now well supported following publication of the EPPPIC results. The absence of universal agreement, though, could be indicative of the current incomplete understanding of the mechanisms underpinning progesterone‐mediated effects in gestational tissues.

## Tocolytics

Tocolytic drugs were among the first agents to be used as potential treatments for PTB. In modern PTB therapeutics, the goal of their use is to delay the progress of spontaneous preterm labor for a period of up to 48 hours. This facilitates the administration of antenatal corticosteroids and magnesium sulfate for fetal benefit as well as the opportunity for in utero transfer to a center with an adequately equipped neonatal unit. Importantly, tocolytics have not been shown to prolong gestation by >48 hours.^43^ In addition, their administration as monotherapy has not demonstrated any improvement in fetal outcomes.^43^ Their mechanism of action mainly targets the contractility pathways. The main class of tocolytic medications for which a role in the regulation of inflammation has been proposed, is the oxytocin receptor (OTR) antagonists, which are discussed below. Two other classes of tocolytics are commonly used: Betamimetics directly suppress contractions by decreasing the activity of myosin light‐chain kinase in myometrial cells. Calcium channel blockers also decrease the activity of myosin light‐chain kinase indirectly by reducing the levels of intracellular calcium. However, neither has been shown to target inflammation, and therefore a more detailed discussion is outside the scope of this review.

### OTR Antagonists

OTR antagonists are commonly used in PTB therapeutics, with an aim to reduce the contractile activity of the uterus in the short term to allow for subsequent administration of fetal optimization medications. Hence, they are considered to be tocolytic agents due to their ability to interfere with the myometrial contraction pathway when binding to the receptor. Specifically, by binding to OTRs in the cell membrane of the myometrial cells, OTR antagonists act to reduce the levels of intracellular calcium by preventing both the release of intracellular calcium and the transfer of extracellular calcium via voltage gated channels.^44^ In addition, the OTR antagonists atosiban and nolasiban have been shown to target inflammatory molecular pathways regulated by NFkB and MAPKs, thereby reducing the expression of procontractile proteins, such as COX‐2.^45^ Atosiban has been the most widely used OTR antagonist to date and the only one outside clinical trials. It was first developed in 1985^46^ and following some promising preliminary studies and a favorable side effect profile, was granted license for administration in European countries in 2000.^47^ However, the current rationale for atosiban administration is to delay labor to allow sufficient time for antenatal corticosteroids to be administered and take effect and has not been shown to prolong delivery by >48 hours. The largest randomized clinical trial to date (APOSTEL‐8) investigating the impact of atosiban in neonatal outcomes in threatened PTB between 30 and 34 weeks is currently in the recruitment phase.^48^

In myometrial cells, MAPKs can be activated secondary to mechanical stretch, resulting in increased c‐fos expression and subsequent activation of the activator protein‐1 cascade.^49^ This in turn stimulates an increased expression of contraction‐associated proteins in the myometrium which acquires a procontractile phenotype.^49^ The OTR antagonist retosiban has been shown to ameliorate the procontractile effects of mechanical stretching in human nonlaboring myometrial explants.^50^ It has also managed to significantly reduce the risk of preterm delivery in cynomologus monkeys.^51^ In these studies, the effects of retosiban have been shown to be at least partly mediated by the OTR. However, the previously suggested crosstalk between the OTR and progesterone receptor raises the biological plausibility that modulation of inflammation could also be involved in the mechanism of action. Unfortunately, the phase III human trials investigating the efficacy and safety of retosiban against placebo (NEWBORN‐1 trial) and against atosiban were terminated in 2020 due to insufficient recruitment.^52^ Similarly, the OTR antagonist barusiban failed to elicit any tocolytic effects within 48 hours when compared to placebo and was therefore never used outside a clinical trial.^53^

Overall, current tocolytic medications have been proven ineffective in prolonging gestation beyond the 48‐hour window and have not demonstrated any benefit for the preterm neonates. Still, their mechanism of action that involves hindering contractions along with a potential to regulate inflammation warranties further research into developing novel tocolytic agents that could achieve a more substantial prolongation of pregnancy along with a decrease in prematurity‐related complications. To this end, the Accelerating Innovation for Mothers initiative has been promoting a Target Product Profile strategy to develop new tocolytic agents that could be used in the management of preterm labor. This also extends to the development of drugs for PTB prevention, where progesterone has been the main medication demonstrating efficacy so far.

## Antibiotics

Intrauterine infection, be it an ascending infection with vaginal bacteria or a transplacental transfer of bacteria from a distant site, has been causally linked to preterm birth.^24^ However, it is the inflammatory reaction to the presence of bacteria in the intrauterine cavity that underpins the activation of myometrial contractions, cervical ripening and dilatation, and membrane rupture.^17^ This means that antibiotics could potentially interfere with the preterm parturition pathway by targeting the bacteria and therefore assisting to a prompt resolution of inflammation rather than directly targeting the inflammatory cascade. The bacteria most commonly associated with PTB can trigger the inflammatory sequelae described earlier in this review to stimulate PTB even in the absence of overt maternal infection both in humans and in animal models.^54,55^ Herein lies a major limitation of antibiotic use: In the absence of symptoms or signs of infection, it is difficult to diagnose infection as the reason for PTB. Even if the suspicion of infection is high in the absence of symptoms, identifying the causative agent to allow for targeted antibiotic treatment is time consuming and often not feasible. Finally, when PTB signs and symptoms with concomitant signs and symptoms of maternal infection often signifies a severe infection, usually in the form of chorioamnionitis, that can trigger a decision for delivery for maternal and/or fetal resuscitation.

Still, antibiotics are currently widely used in the context of different PTB scenarios. However, their role is mainly focused on prevention of fetal/neonatal infection, and they are not considered effective in the prevention or treatment of PTB. In the presence of preterm labor symptoms, antibiotics have been proven to be inefficient both in reducing the rates of PTB and improving neonatal outcomes when fetal membranes are intact.^56^ The landmark ORACLE II trial confirmed the above findings for the antibiotics co‐amoxiclav and erythromycin.^57^ One consideration here is the fact that these antibiotics have been trialed at doses similar to and not exceeding the ones used in nonpregnant women. With physiological changes of pregnancy resulting in increased clearance and overall reduced circulating levels of antibiotics,^58^ a trial of higher doses would not be unreasonable. Still, the Cochrane review showed no effectiveness in prolonging gestation among any of the antibiotics tested including beta‐lactams, macrolides, clindamycin, and metronidazole. In addition, co‐amoxiclav was found to confer detrimental effects to the newborn even at a dose of 375 mg.^57^ This took the form of increased incidence of necrotizing enterocolitis when used as a monotherapy and increased risk of cerebral palsy and neonatal mortality when used in conjunction with erythromycin.^57^ In view of this, antibiotics are currently not used for the treatment of PTB in the absence of preterm premature rupture of membranes (PPROM) unless signs of maternal infection are present, in which case the objective is shifted toward prevention of maternal sepsis rather than prolongation of gestation.

The landscape becomes much more favorable for the use of antibiotics when PPROM is confirmed. Apart from reducing the incidence of subsequent development of chorioamnionitis, antibiotics have also been shown to reduce the risk of delivery within 2 and 7 days.^59^ Still, it is not yet clear whether this is achieved by delaying the onset of spontaneous preterm labor following PPROM or by reducing the need for iatrogenic preterm delivery. Importantly, antibiotics have also been effective in reducing the risk of severe neonatal infection, albeit without completely eradicating this risk.^60^ Similar to the case of intact membranes, the use of co‐amoxiclav in PPROM has been associated with increased incidence of necrotizing enterocolitis in the neonates.^60^

Antibiotics have also been tested in PTB prevention strategies. A common strategy involves treating extrauterine infections with antibiotics, aiming for resolution of infection along with reducing the risk of subsequent PTB. The most common one is bacterial vaginosis, a vaginal infection that has been associated with PTB.^61^ Unfortunately, the antibiotics metronidazole^62^ and clindamycin^63^ have both failed to reduce the incidence of PTB despite achieving satisfactory treatment of bacterial vaginosis. This was also the case when treating infections that have not been associated with PTB, including *Chlamydia trachomatis* ^64^ and *Trichomonas vaginalis*.^65^ Interestingly, a trial in Malawi examined the use of the antibiotic azithromycin for PTB prophylaxis in the absence of any infection. Azithromycin was selected due to its effectiveness against the bacteria most commonly associated with PTB, namely the *Ureaplasma* species. The study showed no reduction in the incidence of PTB with the use of azithromycin.^66^

Overall, despite their widespread use and the deployment of different strategies, antibiotics have had limited success in the prevention and treatment of PTB. This is not surprising as, despite being the most common cause of PTB, infection still accounts for 25% to 40% of the cases.^18^ This means that antibiotic administration should have no effect in the majority of cases. Even when infection is indeed the trigger, it is the subsequent inflammatory response that drives the mechanisms leading to PTB. It is quite plausible that by the time antibiotics are administered, the underlying inflammation has already activated the parturition components resulting in PTB.

## Anti‐Inflammatory Medications

The limited success of tocolytic medications in prolonging delivery and ultimately treating PTB has shifted the attention toward targeting inflammation. Different strategies have been deployed in an attempt to suppress the intrauterine inflammatory reactions representing the hallmarks of inflammation‐associated preterm birth. These can broadly be categorized in 2 main groups: targeting the action of proinflammatory cytokines or their main effector molecules in the context of parturition, prostaglandins.

### Nonsteroidal Anti‐Inflammatory Drugs

Nonsteroidal anti‐inflammatory drugs (NSAIDs) inhibit prostaglandin synthesis by targeting the enzymes COX‐1 and COX‐2.^67^ Prostaglandins are known to play a key role in the initiation of parturition.^15^ They are central in all different components of the parturition pathway including myometrial contractions, cervical ripening and dilatation, and membrane rupture.^15^ This is evident by the fact that prostaglandin levels significantly increase in gestational tissues before the onset of parturition.^68^ In addition, synthetic prostaglandins are widely used for induction of labor.^69^ Human parturition in particular is thought to be mainly mediated by COX‐2, as it is significantly upregulated in the myometrium and fetal membranes before the onset of labor.^70,71^ These enzymes facilitate the production of prostaglandins from arachidonic acid.

Different NSAIDs have been studied in PTB therapeutics. This includes their use both as treatment/tocolytic agents (eg, indomethacin, celecoxib) and as part of prevention strategies (eg, aspirin). Indomethacin, a nonselective COX inhibitor, is the most commonly used among them owing to its anti‐inflammatory and tocolytic effects, along with its ease of administration as an oral preparation. Three small RCTs from the early 80s and late 90s with a total of 102 patients have shown that indomethacin has potential in reducing PTB rates but with no significant difference in the first 48 hours.^72^ This is further supported by a preclinical study in mice, where indomethacin administration managed to significantly decrease the occurrence of lipopolysaccharide (LPS)‐induced PTB.^73^ In addition, indomethacin compares favorably to beta‐mimetics both in PTB reduction and in birth during the first 48 hours.^72^ There are no studies comparing NSAIDs to OTR antagonists for delaying PTB.

Among the other COX inhibitors, rofecoxib, a selective COX‐2 inhibitor, showed significant tocolytic potential in a preclinical study where it reduced the contractile activity of rat myometrial strips in vitro.^74^ However, despite initial evidence from a 2004 RCT in 214 patients showing similar tocolytic effects to magnesium sulfate,^75^ human data from an RCT of 98 women in 2005 showed no effect in PTB rates before 30 weeks and even suggested an increase in both PTB and PPROM before 37 weeks while demonstrating significant fetal side effects.^76^ Celecoxib is another selective COX‐2 inhibitor that has demonstrated a significant dose‐dependent reduction in LPS‐induced PTB rates in mice.^77^ In humans, it has shown similar tocolytic effects to magnesium sulfate among 104 patients in 2007^78^ and with an improved safety profile to indomethacin in a small study of 24 patients in 2002.^79^ Still, there are no large‐scale data to support its use in humans. This is also the case for the nonselective COX inhibitor sulindac, as shown by a small RCT of 69 patients in 1995.^80^ Finally, the selective COX‐2 inhibitor nimesulide managed to arrest contractions in human myometrial strips ex vivo.^81^ This effect was dose dependent and comparable to that of indomethacin.^81^ In a small human trial with 20 women, it shared the same side effects as indomethacin and sulindac.^82^

A major limitation precluding a more widespread testing and use of NSAIDs is their unfavorable safety profile, particularly on the fetal side. NSAID use has been causally linked to premature closure of ductus arteriosus in utero.^83^ Despite being usually transient, this is a potentially serious complication as it can cause pulmonary hypertension in the fetus or the newborn. This effect can be caused at any gestational age, but it becomes much more likely in the third trimester.^84^ Another important consideration is the NSAIDs’ effect on the fetal or neonatal renal function. Prostaglandins are important mediators in renal homeostatic mechanisms, acting as vasodilators to increase renal perfusion.^85^ At the same time, they promote urine production by antagonizing the effects of antidiuretic hormone.^85^ By inhibiting prostaglandin formation, NSAIDs can cause kidney injury. This can take the form of reduced renal blood flow, leading to reduced urine output and subsequent oligohydramnios.^86^ Despite the fact that these side effects are often transient, their potential severity poses an important challenge that could significantly prevent successful recruitment in future trials.

The most notable exception to these safety concerns among the COX inhibitors is aspirin. At low doses of 150 mg or less, aspirin inhibits platelet aggregation while also exhibiting anti‐inflammatory effects. These properties are likely important contributors in the potential of aspirin to prevent preeclampsia, although the precise mechanism is not yet fully understood. Yet aspirin is currently widely used for preventing preeclampsia and has been extensively studied in this context.^87^ This allowed for subsequent secondary analyses using data from the preeclampsia studies to investigate potential associations with PTB reduction.^88^ Indeed, it has been suggested that low‐dose aspirin reduces the risk of spontaneous PTB of <34 weeks with no effect in the overall rate of PTB.^89^ This observation subsequently led to 2 RCTs evaluating aspirin for PTB prevention. The first in 2021, performed in low‐ and middle‐income countries, showed a small but meaningful reduction in PTB rates among nulliparous women.^90^ As there was no discrimination between spontaneous and iatrogenic PTB, it is difficult to assert whether this reduction is indeed reflective or PTB prevention or is secondary to preeclampsia prevention only. The second and most recent study in 2022 (APRIL study) was performed in a high‐income setting (the Netherlands) and showed no reduction in PTB rates among women with a history of previous PTB.^91^ Lower‐than‐expected rates of PTB in the placebo group resulted in the study being underpowered to detect any difference in the observed effect size. Interestingly, no association between aspirin use and premature closure of ductus arteriosus was found across all the preeclampsia and PTB studies. This favorable safety profile along with the cautiously promising findings so far warrant further research determine if aspirin has a role in PTB prevention.

Collectively, due to the moderate to low quality and the limited efficacy demonstrated in human studies, these medications are not currently used in PTB therapeutics.

### Selective Prostaglandin Inhibitors

While NSAIDs inhibit prostaglandin production by blocking COX‐2 and therefore the synthesis of all prostaglandins, a more recent approach involves targeting specific prostaglandins with a central role in parturition mechanistic pathways. The most well studied prostaglandins in this field are prostaglandin E2 (PGE2) and prostaglandin F2a (PGF2a). PGE2 can stimulate myometrial contractions.^92^ PGF2a can also trigger myometrial contraction and at the same time induce matrix metalloproteinase production, thereby facilitating rupture of membranes and cervical ripening.^93,94^

The prostaglandin F2a receptor (FP) antagonist TGH113 was found to prolong gestation in a mouse model of intraperitoneally administered LPS‐induced PTB in a dose‐dependent manner.^95^ Importantly, this led to increased fetal pup survival. Ex vivo experiments showed that this could be mediated by TGH113 exerting a relaxation effect in mouse myometrial strips.^95^ A similar relaxant effect was also elicited in human myometrial explants where contractions were stimulated with oxytocin. However, TGH113 did not manage to prevent PGF2a‐mediated contractions in the same model.^95^ Another FP receptor antagonist named AS604872 also prolonged pregnancy in 2 different models of PTB: the first triggered by systemic LPS administration and the second triggered by functional progesterone withdrawal via mifepristone (RU486) administration.^96^ Ebopiprant or OBE‐022 is another FP antagonist with potential in PTB therapeutics. In a mouse model of mifepristone‐induced PTB, the administration of oral ebopiprant almost doubled the timing of delivery following mifepristone injection.^97^ This effect was significantly enhanced by the coadministration of the calcium channel blocker nifedipine, a drug currently being used as a tocolytic in human preterm labor.^97^ In addition, its safety profile was much more favorable than the NSAID indomethacin. In particular, ebopiprant did not cause ductus arteriosus constriction, renal injury, or inhibition of platelet aggregation, all common side effects of NSAIDs.^97^ As a smooth muscle relaxant, ebopiprant was superior to TGH113 as it demonstrated inhibitory activity against contractions triggered by both oxytocin and PGF2a in human myometrial explants.^97^ These promising findings led to small‐scale trials in humans aiming to evaluate the safety of the drug. Encouragingly, these studies are in agreement about the favorable safety profile of ebopiprant. Specifically, it was found to be well tolerated with minimal side effects either when administered alone^98,99^ or in combination with other medications commonly used in PTB therapeutics, including atosiban, nifedipine, magnesium sulfate, and betamethasone.^100^

Further studies will seek to determine whether prostaglandin receptor antagonists could be used in PTB prevention and treatment strategies. The selectivity and potency of their actions along with the minimal maternal and fetal side effects make these agents a very promising class of medications.

### Cytokine‐Suppressive Anti‐Inflammatory Drugs

Cytokine‐suppressive anti‐inflammatory drugs (CSAIDs) represent a more novel class of drugs compared to NSAIDs. In the past 10 years, several different compounds have been studied as potential modulators of intrauterine inflammatory responses. Yet, so far this has been solely in preclinical models. Two main strategies have been deployed in the design of this drug: broad‐spectrum and selective cytokine/chemokine inhibition.

Broad‐spectrum CSAIDs regulate cytokine signaling by targeting events upstream of cytokine expression. In PTB therapeutics, they specifically target 2 signaling pathways that are crucial in the pathophysiology of inflammation‐induced PTB, namely, the NFkB and MAPK pathways. By inhibiting the NFkB complex via blockade of p60 nuclear translocation, sulfasalazine was able to inhibit the expression of several LPS‐induced proinflammatory cytokines including TNFα and IL‐6 in human fetal membranes ex vivo, albeit while concurrently exhibiting a proapoptotic effect in these tissues.^101^ The I‐kB kinase (IKKB) inhibitors parthenolide and TPCA‐1 were also shown to downregulate NFkB‐dependent TNFα and IL‐6 expression (along with other cytokines/chemokines) in human choriodecidual cells ex vivo, and without affecting the apoptosis pathway.^102^ TPCA‐1 treatment ex vivo was also effective in suppressing IL‐8 and PGE2 levels caused by in utero administration of LPS or *Ureaplasma parvum* in sheep.^103^ In a similar fashion, the MAPK inhibitors U0126, SB202190, and SP600125 did reduce the LPS‐stimulated expression of cytokines, chemokines, and prostaglandins in human placental and membrane explants.^23^ This was also the case for the compound SB239063 in human and ovine fetal membranes.^103^ Importantly, apart from ameliorating inflammation, there is also evidence of CSAIDs potential in PTB treatment. In a mouse model of systemic inflammation–induced PTB using intraperitoneal administration of LPS, the broad‐spectrum chemokine inhibitor somatotaxin achieved a reduction in PTB rates.^104^ This was accompanied by a reduced inflammatory response in maternal gestational and nongestational tissues.^104^ It also conferred the major benefit of live‐born pups with normal weight in the cases were LPS‐induced PTB was prevented.^104^ Another broad‐spectrum chemokine inhibitor named FX125L was tested in a nonhuman primate model as a therapeutic agent for PTB triggered by a choriodecidual inoculation of group B *Streptococcus* (GBS).^105^ While a significant reduction in cytokine/chemokine levels along with a concomitant in PTB rates was observed, FX125L did not prevent GBS colonization of the amniotic cavity.^105^ Subsequently, there was GBS colonization in the fetal lung and development fetal pneumonia.^105^ This highlights the complexity of targeting infection/inflammation in PTB treatment strategies. Prolongation of gestation can be beneficial or detrimental depending on the infectivity of the responsible agent and the status of fetal inflammation. Another important consideration is the potential of these medications to cause a more generalized immunosuppression, as they mostly target upstream molecules of the inflammatory response. This could render mothers and fetuses more susceptible to infections.

A more specific approach involves targeting individual cytokines/chemokines that are central to PTB pathophysiology. This can be achieved either by directly blocking the target cytokine or by blocking the receptor. TNFα, IL‐1β, and IL‐6 are the most well‐studied cytokines in PTB therapeutics. In a mouse model of systemic inflammation induced by intraperitoneal LPS injection, both an IL‐1β and a TNF receptor antagonist showed no effect on preventing PTB or prolonging gestation.^106^ However, anti‐TNF specific antibodies did manage to reduce the rates of PTB along with the expression of key cytokines such as IL‐1β and IL‐6 in similar models.^106^ This was also accompanied by a reduction in fetal death rates.^106,107^ Pretreatment with an antibody targeting the IL‐6 receptor was also shown to significantly reduce the incidence of PTB stimulated by intraperitoneally administered LPS.^108^ In the same study, the IL‐6–specific antibody tocilizumab blocked the production of PGE2 by human amnion explants following LPS stimulation.^108^ Somewhat unexpectedly, a study in pregnant rats around the same time reported that targeting the IL‐6 receptor with an antibody actually exacerbated the proinflammatory effects of the LPS and increased the pup mortality.^109^ IL‐1β blockade has been studied in more specific models of intrauterine inflammation, where the trigger is administered directly to the uterus. The results of this have also been conflicting. The IL‐1β receptor antagonist kineret had no effect in the rate of preterm delivery following an intrauterine injection of LPS.^110^ This result was also the same when LPS was administered intraperitoneally.^110^ At the same time, though, it prevented neonatal brain injury as assessed by the morphology of fetal neurons.^110^ A similar neonatal inflammation‐dampening effect was also reported in sheep, where kineret reduced both the systemic and lung‐specific inflammation facilitating lung maturation.^111^ In a nonhuman primate model of LPS‐induced fetal inflammation, an IL‐1β receptor antagonist was successful in reducing the inflammatory response as measured by cytokine concentrations in the fetal skin.^112^ However, another study using the IL‐1β receptor antagonist rytvela reported some promising findings. In their mouse model of PTB, intrauterine inflammation was induced with direct intrauterine injection of LPS, Lipoteichoic acid (LTA), or IL‐1b.^113^ While all 3 stimulants successfully induced PTB in mice, pretreatment with the IL‐1β receptor antagonist managed to rescue this phenotype by reducing PTB rates.^113^ In addition, it significantly decreased the expression of all the major proinflammatory cytokines in the mouse myometrium.^113^

The available evidence warrants further research into the potential of these medications to be part of PTB prevention or treatment strategies. Future studies need to also evaluate their safety in pregnancy even in the preclinical setting. Nevertheless, this is still a novel and promising field where targeted interventions are guided by deeper understanding of the underlying pathophysiology.

### Immune Response Modulators

Given the central role of inflammation in the pathophysiology of PTB, many substances exhibiting anti‐inflammatory and/or immune‐modulatory properties have been evaluated as potential therapeutic agents. A common characteristic is that these substances have been studied only in the preclinical setting with insights about their action in human pregnancy being extrapolated from in vitro or ex vivo experiments.

A common target in PTB strategies aiming to suppress inflammation is the NFkB pathway. Resveratrol decreases both the levels and the nuclear translocation of p65 thereby inhibiting NFkB transcriptional function.^114^ Daily administration of resveratrol was shown to reduce PTB rates following intracervical administration of LPS in mice.^115^ At the same time, it reduced the expression of TNFα and IL‐1β in peritoneal macrophages.^115^ This anti‐inflammatory effect was also produced in human placental and fetal membrane explants where resveratrol downregulated the expression of LPS‐induced TNFα, IL‐6, and IL‐8.^116^ Another potential therapeutic agent targeting the NFkB pathway is N,N‐dimethylacetamide. By blocking p65, N,N‐dimethylacetamide reduced the placental inflammatory response triggered by LPS in a mouse model of PTB.^117^ The expression of TNFα, IL‐1β, and IL‐6 was reduced, while that of IL‐10 was increased.^117^ This was also accompanied by a reduction in PTB incidence.^117^ The antioxidant N‐acetylcysteine was reported to significantly prolong the timing of delivery following intraperitoneal LPS administration in mice.^118^ At the same time, it protected against inflammation‐induced fetal injury via oxidative stress reduction.^118^ A similar protective effect conferring a decrease in PTB rates along with a reduced incidence of maternal inflammation and fetal white matter injury in pups was found when LPS was administered directly to the uterus following laparotomy.^119^ Melatonin and folic acid have also been known to exert anti‐inflammatory functions by at least partly suppressing the NFkB pathway. Subcutaneous melatonin prevented PTB and increased pup survival in a mouse model of intraperitoneal LPS administration, while decreasing myometrial prostaglandin and TNFα levels.^120^ This was also confirmed in a more recent study, where melatonin reduced PTB rates while also conferring a neuroprotective effect in the pups by decreasing neuroinflammation and subsequent brain injury.^121^ In a similar fashion, folic acid pretreatment reduced LPS‐induced PTB and fetal death.^122^ The reduction was achieved at least partly by suppressing NFkB‐mediated placental inflammation.^122^ Finally, the anti‐inflammatory prostaglandin 15d‐PGJ2 was found to inhibit NFkB activity in human explants from amnion and myometrial tissues.^123^ In addition, it could also regulate COX‐2 activity.^123^ In a mouse model of intrauterine LPS‐induced PTB, coadministration of 15d‐PGJ2 prolonged the timing of delivery while reducing pup mortality.^124^

A more recent therapeutic target in PTB pharmacology involves the inhibition of the TLR‐4 pathway.^125^ This is the receptor of LPS, the most common substance used to induce PTB in preclinical murine models. Antagonizing this receptor significantly reduced the expression of proinflammatory cytokines/chemokines including TNFα and IL‐8 and prostaglandins including PGE2 and PGF2a following LPS challenge in a nonhuman primate model.^126^ This was accompanied by a reduction in the contractile activity of the myometrium in vivo.^126^ In mice, TLR4 blockade reduced the incidence of LPS‐induced preterm delivery along with decreasing systemic maternal and placenta inflammation as measured by the expression of immune cells.^127^ Another TLR4 antagonist, naloxone, managed to both protect from LPS‐induced PTB, but to also reduce the levels of TNFα, IL‐1β, and IL‐6 in mouse gestational tissues.^128^ Rosiglitazone also decreased PTB rates while reducing oxidative stress in mouse decidual and peritoneal macrophages following LPS‐induction.^129^ More recently, the TLR4 antagonist naltrexone was found to inhibit PTB and pup demise in a mouse model where PTB was triggered by carbamyl platelet‐activating factor, a synthetic analogue of platelet‐activating factor, which is a potent NFkB activator in myometrial tissues.^130^ This was accompanied by a significant reduction of the levels of IL‐6 and IL‐1β in the myometrium and decidua.^130^

Another area that has recently attracted attention in the PTB pathophysiology with potential therapeutic implications is that of the vaginal microbiome. Differential dominance of different types of lactobacilli in the vagina identifies risk profiles when it comes to PTB.^131^ Specifically, dominance of *Lactobacillus crispatus* appears to confer a protective effect whereas dominance of *Lactobacillus iners* increases the risk for PTB.^132,133^ Although the mechanisms behind these associations are still evaluated, a recent study has shown that immune dysregulation can occur depending on the microbial composition in the vagina and this can lead to PTB.^134^ *L crispatus* was not shown to trigger inflammation and could potentially be used as a therapeutic agent aiming at restoring this dysregulation and preventing PTB.^134^ In addition, a preclinical study reported decreased PTB rates in mice given intrauterine LPS injections when a prior intraperitoneal injection of a supernatant from *Lactobacillus rhamnosus* GR‐1 was applied.^135^ At the same time, the *L rhamnosus* supernatant reduced the levels of LPS‐induced IL‐1β, IL‐6, and TNFα along with other cytokines in gestational tissues and maternal plasma.^135^

Finally, recent studies have evaluated potential therapeutic agents that are not known to target mechanistic pathways directly implicated in the PTB pathophysiology. A common characteristic of these agents is that their exhibit anti‐inflammatory properties. Some promising data have been reported about endothelin‐1 (ET‐1). ET‐1 causes smooth muscle contraction resulting in vasoconstriction.^136^ In addition, it can cause upregulation of proinflammatory cytokines via its receptor.^136^ In a mouse model of systemic inflammation induced by intraperitoneal administration of LPS, inhibiting the expression of ET‐1 was shown to significantly reduce the incidence of preterm delivery.^137^ In this model, rescue of the LPS‐induced PTB phenotype was achieved both by limiting the synthesis of ET‐1 and by blocking its receptor.^137^ In a further study on the same model, the ET‐1 receptor antagonist BQ‐123 also reduced the rates of PTB.^138^ At the same time, it decreased the expression of placental TNFα and IL‐1β, while increasing the expression of the anti‐inflammatory cytokine IL‐10.^138^ Importantly, in this model the administration of BQ‐123 was after the LPS stimulation, while the treatment in such as models is often administered before and after LPS. This could signify therapeutic potential even after a diagnosis of PTB is made. Inhibition of sphingosine kinase, a downstream molecule activated following ET‐1 receptor ligation, was also found to block LPS‐induced PTB while reducing placental inflammation as measured by a decreased expression of TNFα, IL‐1β, and IL‐6.^139^

Substances involved in lipid metabolism have also been studied in PTB therapeutics. Mice genetically engineered to produce high levels of polyunsaturated omega‐3 fatty acids have some levels of protection against inflammation‐induced PTB, as shown by the lower incidence of preterm delivery following intravaginal LPS administration.^140^ In addition, exogenous administration of the omega‐3 fatty acid resolving E3 rescued the PTB phenotype of wild‐type mice treated with LPS.^140^ Mechanistically, it is likely that this protective effect is due to the anti‐inflammatory potential of omega‐3, as shown by the decreased levels of IL‐1β and IL‐6 along with the lower numbers of macrophages in the mutant mice.^140^ Lipoxin A4 has been shown to reduce the expression of IL‐6 and IL‐8 following LPS stimulation in human myometrial explants in vitro.^141^ In a mouse model of intrauterine LPS‐induced PTB, pretreatment with lipoxin A4 significantly reduced pup mortality.^142^ This was despite the fact that lipoxin A4 had no effect on the timing of delivery as it did not affect PTB rates.^142^ Statins represent another group of drugs with therapeutic potential in PTB, due to their anti‐inflammatory and antioxidative effects. Pretreatment with simvastatin was found to decrease the incidence of preterm delivery following ultrasound‐guided intrauterine LPS injection.^143^ This was accompanied by an anti‐inflammatory effect both in gestational tissues and systemically as measured by serum IL‐6 levels.^143^ A similar effect was reported in human myometrial cells treated with LPS in vitro, where simvastatin reduced the expression of IL‐6 and IL‐8 while increasing the expression of IL‐10 and IL‐13, thereby suppressing inflammation.^143^

Collectively, many different immune‐modulatory agents have demonstrated therapeutic potential in the preclinical setting. This promising novel field will still require further investigations before treatment strategies can be tested in humans.

## Concluding Remarks

PTB remains the worldwide leading cause of perinatal, neonatal and child mortality, and morbidity. Despite the vast scale of the problem, there is still an apparent lack of successful therapeutic strategies to tackle PTB. Most agents used currently have been produced based on knowledge from the 1960s and 1970s, indicating a lack of development in the field and a scarcity of newer medications coming to the market. This is further intensified by the fact that most phase II/III clinical trials have yielded negative results, precipitating a vicious cycle of underfunding and underdelivering.

While the global health and socioeconomic impact of prematurity along with the paucity of therapeutic options remain largely unchanged, scientific advances have broadened the current understanding of the mechanisms underpinning the complex pathophysiology of PTB. This enhanced knowledge, along with the limited success of agents traditionally used in PTB therapeutics that were aiming at stopping uterine contractions, have shifted the attention toward targeting intrauterine inflammation. With infection being the leading cause of PTB and inflammation driving the molecular events leading to PTB and fetal injury, this approach is already yielding promising results that are being used in the development of novel therapeutic agents and treatment strategies. Still, careful consideration should be given to a number of factors including efficacy, side effects for both the mother and the fetus in the short and long term, selectivity in action and optimal route of administration. Concurrent progress in the field of diagnostics should also allow correct identification of women and babies that would benefit from such treatment options. Overall, the research community is now as well equipped as ever to achieve further advances that will allow for effective prevention and treatment strategies to be developed. There is an urgent need among all stakeholders to realize previous shortfalls and current potential and adopt a collaborative approach to substantially reduce the impact of PTB.

## Conflicts of Interest

I.P. has no competing interests to declare. S.J.S. has received research grants paid to the institution from Wellcome Trust, Scottish Chief Scientist Office, National Institute of Healthcare Research, and Tommy's during the course of this study. S.J.S. has received honoraria (paid to institution) from Hologic for research talks. S.J.S. has received fees for consultancy (paid to institution) from Natera on products for preterm labor treatment.

## Data Sharing

Data used to inform this review is cited within the text. Any data requests should be directed to the authors of the original papers.

## Funding

I.P. is funded by a National Institute for Health Research (NIHR) Academic Clinical Fellowship. S.J.S. is funded by a Wellcome Trust Clinical Career Development Fellowship (209560/Z/17/Z).

**Table 1:** Agents Studied in PTB Therapeutics and Their Proposed Mechanisms of Targeting Inflammation

Agent	Inflammation Target	References
Progesterone	Proinflammatory cytokine reduction	^27,28^
17‐OHPC	Proinflammatory chemokine reduction	
	PR antagonizes NFkB activation of COX‐2	
Atosiban	Reduction of NFkB and MAPK‐mediated expression of COX‐2	^45^
Nolasiban		
Beta‐lactams	Killing or inhibition of growth of bacteria	^56,57,59,61‐63,66^
Macrolides		
Clindamycin		
Metronidazole		
Indomethacin	COX‐1 and COX‐2 inhibition	^72,73,80,89‐91^
Sulindac		
Aspirin		
Rofecoxib	Selective COX‐2 inhibition	^74‐77,81^
Celecoxib		
Nimesulide		
TGH113	Prostaglandin F2a receptor antagonists	^95‐100^
AS604872		
Ebopiprant		
Sulfasalazine	NFkB inhibition	^101‐103,115‐124^
Parthenolide	Proinflammatory cytokine reduction	
TPCA‐1		
Resveratrol		
DMA		
NAC		
Melatonin		
Folic acid		
15d‐PGJ2		
U0126	MAPK inhibition	^23,103^
SB202190		
SP600125		
B239063		
Somatotaxin	Broad‐spectrum chemokine inhibitor	^104,105^
FX125L		
Naloxone	TLR4 antagonists	^128,129,130^
Rosiglitazone	Proinflammatory cytokine reduction	
Naltrexone		
ET‐1 inhibitor	Proinflammatory cytokine reduction	^137,139,141,142^
SphK inhibitor		
Lipoxin A4		
BQ‐123	Proinflammatory cytokine reduction	^138,143^
Simvastatin	Antiinflammatory cytokine increase	

COX, cyclooxygenase; DMA, N,N‐dimethylacetamide; ET‐1, endothelin‐1; MAPK, mitogen‐activated protein kinase; NAC, N‐acetylcysteine; NFkB, nuclear factor kappa B; 17‐OHPC, 17‐hydroxyprogesterone caproate; PTB, preterm birth; SphK, sphingosine kinase; TLR4, toll‐like receptor 4.
